# Identification of Secondary Metabolite Gene Clusters in the *Pseudovibrio* Genus Reveals Encouraging Biosynthetic Potential toward the Production of Novel Bioactive Compounds

**DOI:** 10.3389/fmicb.2017.01494

**Published:** 2017-08-18

**Authors:** Lynn M. Naughton, Stefano Romano, Fergal O’Gara, Alan D. W. Dobson

**Affiliations:** ^1^School of Microbiology, University College Cork, National University of Ireland Cork, Ireland; ^2^Division of Microbial Ecology, Department of Microbiology and Ecosystem Science, University of Vienna Vienna, Austria; ^3^School of Biomedical Sciences, Curtin University, Perth WA, Australia; ^4^BIOMERIT Research Centre, School of Microbiology, University College Cork, National University of Ireland Cork, Ireland

**Keywords:** symbiont, PKS, NRPS, antibiotic resistance, bioactive compounds

## Abstract

Increased incidences of antimicrobial resistance and the emergence of pan-resistant ‘superbugs’ have provoked an extreme sense of urgency amongst researchers focusing on the discovery of potentially novel antimicrobial compounds. A strategic shift in focus from the terrestrial to the marine environment has resulted in the discovery of a wide variety of structurally and functionally diverse bioactive compounds from numerous marine sources, including sponges. Bacteria found in close association with sponges and other marine invertebrates have recently gained much attention as potential sources of many of these novel bioactive compounds. Members of the genus *Pseudovibrio* are one such group of organisms. In this study, we interrogate the genomes of 21 *Pseudovibrio* strains isolated from a variety of marine sources, for the presence, diversity and distribution of biosynthetic gene clusters (BGCs). We expand on results obtained from antiSMASH analysis to demonstrate the similarity between the *Pseudovibrio*-related BGCs and those characterized in other bacteria and corroborate our findings with phylogenetic analysis. We assess how domain organization of the most abundant type of BGCs present among the isolates (Non-ribosomal peptide synthetases and Polyketide synthases) may influence the diversity of compounds produced by these organisms and highlight for the first time the potential for novel compound production from this genus of bacteria, using a genome guided approach.

## Introduction

The global threat of antimicrobial resistance (AMR) has reached a crisis point with both common and life-threatening infections becoming increasingly untreatable. According to the Centers for Disease Control and Prevention, at least 2 million people become infected with antibiotic resistant bacteria each year in the United States, with 23,000 deaths occurring as a direct result of these infections (Centers for Disease Control and Prevention, 2013). This number is estimated to rise to a staggering 10 million deaths by 2050 (O’Neill, 2014). The onslaught of this threat became ever more apparent following the emergence of a pan-resistant strain of *Klebsiella pneumoniae* in August 2016, which was resistant to every available antibiotic in the United States (26 in total) (Chen et al., 2017).

The rise in incidences of AMR and decline in drug discovery from traditional sources (e.g., terrestrial plants and microbes) has led to a rapid shift in focus toward marine-derived natural products. Given that the marine ecosystem represents 95% of earth’s biosphere, it is perhaps not surprising that our oceans contain a veritable “treasure trove” of diverse chemical compounds. Marine sponges (*Porifera*) in particular have gained notoriety as prolific producers of chemically diverse bioactive compounds (Faulkner, 2002; Laport et al., 2009; Hertiani et al., 2010; Turk et al., 2013). In 2014 alone, 283 novel compounds were reported from the phylum Porifera (Blunt et al., 2014). As sessile organisms, sponges are highly susceptible to predation by fish and other invertebrates. As a result, they have developed a sophisticated armory of defensive chemicals to deter predators and prevent growth on their surfaces by competitive species (fouling organisms) (Mol et al., 2009; Hertiani et al., 2010). The cytotoxicity of these compounds is particularly potent in habitats such as coral reefs where competition and predation are intense (Proksch, 1994). The chemical diversity of these bioactive compounds is immense, ranging from nucleosides, cyclic peptides and fatty acids to amino acid derivatives, possessing antibacterial, anticancer, anti-inflammatory, and anti-viral properties to name but a few (Sagar et al., 2010; Villa and Gerwick, 2010; Frota et al., 2012; Mehbub et al., 2014). However, in the last number of years mounting evidence suggests that microbial-symbionts of sponge species are in fact the true producers of these bioactive molecules (Donadio et al., 2007; Piel, 2009).

These bioactive molecules are often synthesized using building blocks derived from primary metabolism by a series of proteins encoded by genes localized close to each other in bacterial genomes, forming a so-called biosynthetic gene cluster (BGC). A BGC represents both a biosynthetic and an evolutionary unit. In some cases, it is possible to roughly infer the molecular backbone of the products synthesized from these clusters by analyzing the specific signatures in the amino acid sequences of their associated biosynthetic proteins. BGCs are commonly classified based on their product as; saccharides, terpenoids, ribosomally synthesized and post-translationally modified peptides (RiPPs), non-ribosomal peptide synthetases (NRPSs), and polyketide synthases (PKSs). NRPS and PKS BGCs in particular have gained much attention in recent years. Non-ribosomal peptides and polyketides are commercially valuable molecules, synthesized in a modular fashion by large multi-enzyme complexes, and account for the majority of structurally diverse and clinically relevant known bioactive natural products (Doroghazi and Metcalf, 2013; Xiong et al., 2013). These enzyme complexes consist of functional units known as modules, which contain at least three essential domains; (i) a catalytic domain responsible for selection of a specific monomer (ii) a carrier protein domain which facilitates attachment of the monomer after thioesterification and (iii) a second catalytic domain which functions in chain elongation (Fischbach and Walsh, 2006). In a typical NRPS module, these domains are represented by; a condensation domain (C), an adenylation domain (A) and a peptidyl carrier protein (PCP) domain (also known as a thiolation domain). Analysis of the ten amino acids which line the binding pocket of the A domains, facilitates prediction of the specific monomer incorporated at this site. This allows for the prediction of the amino acid backbone structure of the resulting peptide (Finking and Marahiel, 2004). A typical PKS module will minimally consist of a ketosynthase (KS) domain, an acyltransferase (AT) domain and an acyl carrier protein (ACP) domain (Fischbach and Walsh, 2006; Donadio et al., 2007). PKSs can be further classified into three types that differ in the organization of their catalytic domains (Shen, 2003). Type I PKSs consist of multi-domain polyproteins which can be classified as (i) modular biosynthetic complexes, consisting of a series of enzymatic domains for each chain extension and modification step or (ii) iterative, wherein a single set of enzymatic domains are reused several times during polyketide biosynthesis (Fischbach and Walsh, 2006). Type II PKSs consist of discrete, separable proteins which form putative multienzyme complexes (Shen, 2003). Type III PKSs, also known as chalcone synthase-like PKSs consist of a simple homodimeric architecture and function essentially as condensing enzymes (Austin and Noel, 2003). Each module associated with these systems may additionally contain tailoring domains such as heterocyclization and epimerisation (E) domains (in the case of NRPS modules) or β-ketoreductase (KR) and dehydrogenase (DH) domains (as in the case of PKS modules) which contribute to the overall structural diversity of the resulting final products (Liou and Khosla, 2003; Donadio et al., 2007). In addition, the structural and functional similarities shared between NRPS and PKS clusters can result in hybrid peptide-polyketide products, offering an even greater variety of secondary metabolites (Miller et al., 2002; Liu et al., 2004).

Traditionally, the discovery of secondary metabolites was based on a bioassay-guided approach involving the cultivation of microorganisms, chemical extraction of the metabolites produced and final structure elucidation. In the past, this approach facilitated the discovery of many valuable chemicals, however, nowadays, too often it results in the rediscovery of known metabolites, leading to a dramatic reduction in the number of new molecules identified (Reen et al., 2015). Therefore, in the last number of years, both analytical and bioinformatic based approaches have been optimized to minimize the likelihood of re-discovery of the same products, thereby increasing the chances of de-replication. Considering the dramatic increase in the number of bacterial genomes which have become available in recent years, the first step in identifying “talented” microbes (which may produce biomolecules with novel bioactivities) lies in genome sequence analysis and subsequent characterization of the BGCs which they encode. This approach facilitates the identification of genomic entities likely responsible for the production of these new molecules.

Bacteria belonging to the genus *Pseudovibrio* have been repeatedly detected worldwide from an array of marine invertebrates, particularly sponges (Crowley et al., 2014). They have often been found to be the most abundant isolates in the culturable bacterial fraction obtained from phylogenetically distinct marine sponges (Webster and Hill, 2001; Muscholl-Silberhorn et al., 2008; Esteves et al., 2013; Bauvais et al., 2015). Bacteria belonging to this genus are metabolically versatile alphaproteobacteria (Bondarev et al., 2013; Romano et al., 2016) and in recent years, have gained much attention, being considered an attractive source of potentially new bioactive compounds (Crowley et al., 2014). *Pseudovibrio* species have been shown to display bioactivity against a number of prominent human pathogens, including *Clostridium difficile*, *Escherichia coli*, *Salmonella enterica* serovar Typhimurium and Methicillin-resistant *Staphylococcus aureus* (O’Halloran et al., 2011), as well as against a number of fish pathogens (Harrington et al., 2014). However, in most cases the recurrent identification of inhibition properties toward pathogenic bacteria associated with these organisms has been linked to the production of the tropolone derived, sulfur-containing antibiotic, tropodithietic acid (TDA) (Harrington et al., 2014). Very few new secondary metabolites have been characterized from *Pseudovibrio* (Sertan-de Guzman et al., 2007; Nicacio et al., 2017). Moreover, even though molecular approaches have suggested the presence of NRPS and PKS BGCs in *Pseudovibrio* isolates (Esteves et al., 2013; Alex and Antunes, 2015), the diversity and distribution of these BGCs within the genus has still not been widely reported. In the current study, we interrogated 21 publicly available *Pseudovibrio* genomes for the presence of BGCs. We report their distribution and similarity within the genus and with BGCs characterized from other organisms, revealing the uniqueness and variability of these gene clusters within the *Pseudovibrio* genus.

## Materials and Methods

### 16S rRNA Phylogenetic Tree Construction

16S rRNA sequences from 21 publically available *Pseudovibrio* genomes were downloaded from the NCBI database (NCBI Resource Coordinators, 2017). 16S rRNA sequences from *Streptomyces tumescens*; Km-1-1; AF346482 and *Streptomycetaceae*; SR 179c; X95470 were downloaded from the Ribosome Database Project for use as outgroups in the analysis (Cole et al., 2014). Sequences were aligned using ClustalW from MEGA7.0 (Kumar et al., 2016) and trimmed to an equal length. The evolutionary history was inferred using the Neighbor-joining method (Saitou and Nei, 1987) with 1000 bootstrap replicates using MEGA7. Evolutionary distances were computed using the Maximum Composite Likelihood method (Tamura et al., 2004).

### Identification of BGCs in the *Pseudovibrio* Genomes

All genome assemblies were recovered from the NCBI database and annotated using Prokka v1.10 (Seemann, 2014). The antibiotics and Secondary Metabolites Analysis Shell, antiSMASH v3.0 (Weber et al., 2015) was used to predict BGCs among the *Pseudovibrio* isolates under study, using default parameters and incorporation of the ClusterFinder algorithm. The annotation of the genomes was then screened to identify additional genes encoding proteins potentially involved in the synthesis of polyketides and non-ribosomal peptides missed during the antiSMASH analysis. The annotation of the SnoaL-like protein was confirmed by scanning the amino acid sequences with the standalone version of Interpro v5.23-62.0 (Jones et al., 2014).

### Similarity Networks

The genbank files of the BGCs predicted by antiSMASH were recovered and the amino acid sequences of the biosynthetic genes involved in secondary metabolite production were extracted using custom Python scripts, by looking for all the CDS that were classified by antiSMASH to be involved in secondary metabolite synthesis (*sec_met* qualifier in the genbank file). Sequences were then concatenated and used to produce global alignments via VSEARCH v1.1.1 (Rognes et al., 2016). Only sequences that shared an identity of 35% were considered for further analysis. Results were then collected and a coverage index was calculated as the ratio between the alignment length (L_al) minus the number of opened gaps (Gaps), divided by the length of the longest sequence between the query and the target sequence [L_long; coverage = (L_al – Gaps)/L_long]. Only alignments >10% were considered for further analysis. Data was then imported in Cytoscape v3.2.1 (Shannon et al., 2003) and visualized as a similarity network based on a perfuse force directed layout using the percent identity as a weight for the length of the spring. Similarity networks between the *Pseudovibrio* BGCs and known BGCs were performed by extracting the amino acid sequences of the biosynthetic genes involved in secondary metabolites production from the most similar cluster identified by antiSMASH, after downloading the BGCs from the Minimum Information about a BGC database (Medema et al., 2015) and annotating them using the standalone version of antiSMASH v3.0. For the BGCs involved in the synthesis of O-antigen-like compounds the amino acid sequences of the known biosynthetic genes were manually downloaded from the MIBiG portal. Sequences were then aligned as reported above, not using any similarity or coverage threshold. Network similarity was then visualized using Cytoscape as previously indicated.

### Phylogenetic Analysis of the (i) AMP-Binding Domains of NRPS Clusters and the (ii) KS Domains of PKS Clusters

Domain architectures of the NRPS and PKS clusters were extracted from the antiSMASH annotation using custom Python scripts recovering all domain annotations (qualifier *domain*) for the gene having *aSDomain* as a feature type in the genbank file. Gene orientation was then manually inspected. Version 1.3 of the MIBiG repository was downloaded and all bacterial BGCs were retained. Sequences were annotated with the standalone version of antiSMASH and all AMP-binding and KS domain amino acid sequences were extracted. Redundancy in the datasets was reduced by clustering the sequences using CD-HIT (Li and Godzik, 2006) with a similarity threshold of 95%. These reduced datasets were then combined with the sequences of the domains identified in the *Pseudovibrio* genomes, and an alignment was performed using Kalign v2.04 (Lassmann and Sonnhammer, 2005). Conserved aligned regions were then retained using trimAl v1.4.rev15 (Capella-Gutierrez et al., 2009) using the *automated1* flag and phylogenetic trees were constructed using FastTree v2.1.7 SSE3 (Price et al., 2009) and the following parameters: *-slow -wag -gamma –bionj*. Trees were then visualized and annotated using Figtree^1^ and subtrees containing *Pseudovibrio* sequences were extracted using Dendroscope (Huson et al., 2007).

## Results

### Strain Information, General Genome Features and Biosynthetic Gene Cluster Diversity within the *Pseudovibrio* Genus

The publically available genomes of 21 *Pseudovibrio* isolates were obtained from the National Centre for Biotechnology Information (NCBI Resource Coordinators, 2017) and interrogated for the presence of gene clusters with putative secondary metabolite biosynthetic capabilities. The genomes were obtained from strains isolated from a number of marine sources from different geographic locations. The majority of the isolates are marine sponge derived; *Pseudovibrio* species with an ‘AD’ designation are isolates of the marine sponge *Axinella dissimilis*, sourced by our group from the south coast of Ireland and known to display antimicrobial activity against a number of prominent human pathogens (O’Halloran et al., 2011; Romano et al., 2016). *Pseudovibrio* sp. AB134 (MIEL01 in this study) was isolated from the marine sponge *Arenosclera brasiliensis* in Brazil (Nicacio et al., 2017), *Pseudovibrio* sp. JE062 was isolated from the marine sponge *Mycale laxissima* in the Florida Keys (Enticknap et al., 2006) and *Pseudovibrio* sp. POLY-S9 was isolated from the marine sponge *Polymastia penicillus* from the Atlantic coast of Portugal (Alex and Antunes, 2015). Isolates from other marine sources include *Pseudovibrio ascidiaceiocola* F423 isolated from an ascidian in Japan (Fukunaga et al., 2006), *P. denitrificans* DSM17465 and JCM12308 isolated from seawater in Nanwan Bay, Taiwan (Shieh et al., 2004), *Pseudovibrio* sp. FO-BEG1 was isolated from a *Beggiatoa* sp. enrichment culture, originally obtained from a black band diseased coral collected off the coast of Florida (Bondarev et al., 2013), *P. hongkongenesis* MCCC 1K00451 (UST20140214-015B) (Xu et al., 2015) and *P. stylochi* MCCC 1K00452 (UST20140214-052) (Zhang et al., 2016) both isolated from the same marine flatworm specimen (*Stylochus* sp.) in Hong Kong and *Pseudovibrio* sp. Tun.PHSC04-5.I4 isolated as part of a study on microbial community genomics and transcriptomics in extreme cold, from Lake Vida (a hypersaline lake) in Antarctica (Murray, 2013).

The genome size of the isolates ranged from 3.68 Mb (*P. stylochi* MCCC 1K00452) to 6.55 Mb (*Pseudovibrio* sp. POLY-S9) with an average genome size of 5.72 Mb. The lowest G + C content was 45.2%, observed in the genome of *Pseudovibrio* sp. AD26; the highest G + C content was 52.5%, detected in the genome of *Pseudovibrio* sp. FO-BEG1. Amongst the available *Pseudovibrio* genomes, only that of strain FO-BEG1 can be considered complete, consisting of two replicons; one chromosome of 5.5 Mbp and a large plasmid of 0.4 Mbp. All the others are divided in a number of fragments, ranging from 8 in *Pseudovibrio* sp. Tun.PHSC04-5.I4 to 286 in *Pseudovibrio* sp. JCM19062 (**Table 1**).

**Table 1:** General genome features of *Pseudovibrio* strains used in this study.

Species	Strain	Length	GC	Fragments	GenBank Id	Isolation source	Reference
*Pseudovibrio ascidiaceicola*	DSM 16392 (F423)	5845495	49.76	45	FOSK00000000.1	Ascidian	Fukunaga et al., 2006
*Pseudovibrio axinellae*	AD2	5126200	50.3	162	LMCB00000000.1	Marine sponge	Romano et al., 2016
*Pseudovibrio* sp.	AD5	6061014	49.87	66	LMCH00000000.1	Marine sponge	Romano et al., 2016
*Pseudovibrio* sp.	W64 (AD8)	5935921	50.13	49	LMCI00000000.1	Marine sponge	Romano et al., 2016
*Pseudovibrio* sp.	AD13	6001312	50.64	36	LMCC00000000.1	Marine sponge	Romano et al., 2016
*Pseudovibrio* sp.	AD14	6201736	50.02	57	LMCD00000000.1	Marine sponge	Romano et al., 2016
*Pseudovibrio* sp.	W74 (AD15)	6190724	50.29	64	LMCJ00000000.1	Marine sponge	Romano et al., 2016
*Pseudovibrio* sp.	AD26	6181400	45.17	159	LMCE00000000.1	Marine sponge	Romano et al., 2016
*Pseudovibrio* sp.	WM33 (AD30)	5745729	51.02	159	LMCK00000000.1	Marine sponge	Romano et al., 2016
*Pseudovibrio* sp.	AD37	5875058	50	224	LMCF00000000.1	Marine sponge	Romano et al., 2016
*Pseudovibrio* sp.	AD46	6124061	49.79	85	LMCG00000000.1	Marine sponge	Romano et al., 2016
*Pseudovibrio*	DSM 17465	6080381	52.2	36	FPBD00000000.1	Seawater	Shieh et al., 2004
*denitrificans*	JCM 12308	6053738	52.2	94	BAZK00000000.1	Seawater	Shieh et al., 2004
*Pseudovibrio hongkongensis*	MCCC 1K00451 (UST20140214-015B)	3746600	51.68	39	LLWC00000000.1	Marine flatworm	Xu et al., 2015
*Pseudovibrio stylochi*	MCCC 1K00452 (UST20140214-052)	3682052	46.16	46	LLWE00000000.1	Marine flatworm	Zhang et al., 2016
*Pseudovibrio* sp.	MIEL01 (AB134)	5975630	52.1	39	MIEL00000000.1	Marine sponge	Nicacio et al., 2017
*Pseudovibrio* sp.	FO-BEG1	5916782	52.5	2	CP003147.1; CP003148.1	*Beggiatoa* sp. co-culture	Bondarev et al., 2013
*Pseudovibrio* sp.	JCM 19062	4607025	51.02	286	BAXV00000000.1	Unknown	Unpublished
*Pseudovibrio* sp.	JE062	5717078	52.15	53	ABXL00000000.1	Marine sponge	Enticknap et al., 2006
*Pseudovibrio* sp.	POLY-S9 (PPL9)	6559398	50.57	163	LCWX00000000.1	Marine sponge	Alex and Antunes, 2015
*Pseudovibrio* sp.	Tun.PHSC04-5.I4	6549844	50.49	8	FNLB00000000.1	Hypersaline lake	Murray, 2013

**FIGURE 1:**
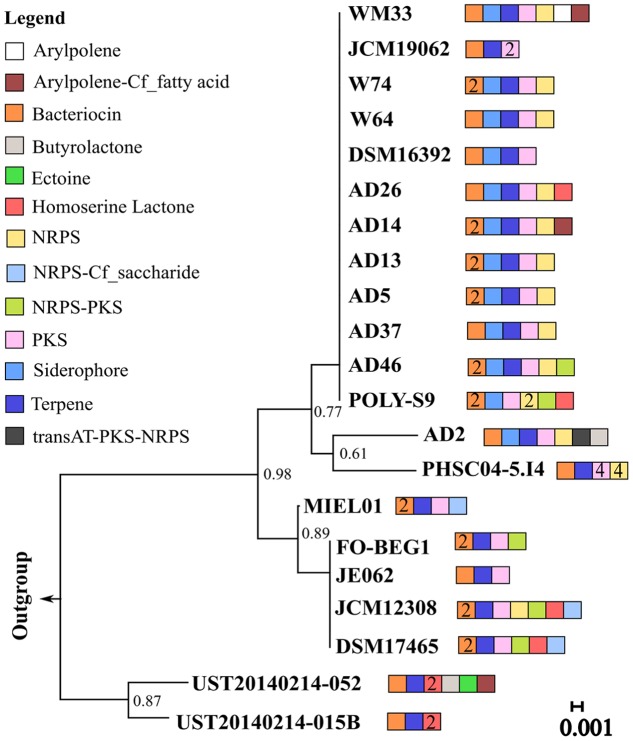
16S rRNA gene phylogeny and biosynthetic gene cluster distribution among members of the *Pseudovibrio* genus. 16s rRNA sequences from 21 *Pseudovibrio* strains and from *Streptomyces tumescens*; Km-1-1; AF346482 and *Streptomycetaceae*; SR 179c; X95470 (outgroups) were aligned using ClustalW from MEGA7.0 and trimmed to an equal length. The phylogenetic tree was constructed according to the neighbor-joining method with 1000 bootstrap replicates using MEGA7. The tree is drawn to scale, with branch lengths in the same units as those of the evolutionary distances used to infer the phylogenetic tree. *Pseudovibrio* strain designations are indicated on each branch of the tree. Colored boxes represent different biosynthetic gene clusters (BGC) types. Where present, numerical values within these boxes correspond to the number of that particular BGC type identified in the adjacent *Pseudovibrio* strain.

Genomic analysis revealed the presence of 129 BGCs distributed among the 21 *Pseudovibrio* isolates examined (**Supplementary Table S1** and **Figure 1**). These included gene clusters for the production of aryl polyene and related products (x4), bacteriocins (x31), butyrolactone (x2), ectoine (x1), homoserine lactone (x8), siderophores (x12) and terpenes (x20). The most abundant classes of BGCs were NRPS, PKS and NRPS-PKS hybrid clusters (51 inclusive; **Figure 1** and **Supplementary Table S1**). Considering their abundance in the genus and the notoriety of structurally and functionally diverse natural products associated with these particular BGC types, we decided to focus the main body of our work on the analysis of the PKS, NRPS and NRPS-PKS hybrid clusters identified in the *Pseudovibrio* isolates.

**FIGURE 2:**
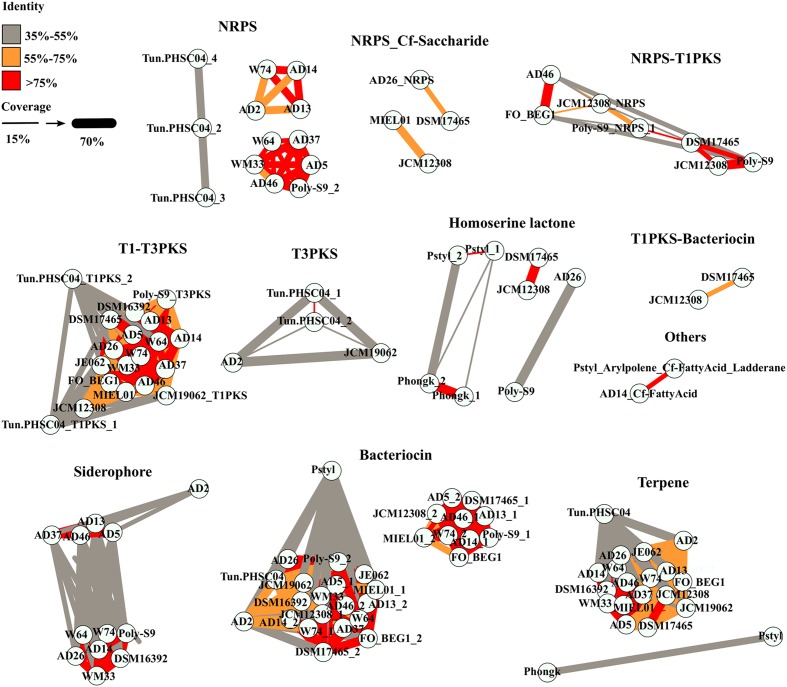
Similarity between the BGCs detected in the *Pseudovibrio* genomes. Each node represents the concatenated amino acid sequences of the biosynthetic genes extracted from the BGCs predicted by antiSMASH. Thickness of the edges are proportional to a coverage index, calculated as the ratio between the alignment length (L_al) minus the number of opened gaps (Gaps), divided by the length of the longest sequence between the query and the target (L_long; coverage = (L_al – Gaps)/L_long). Colors of the edges indicate the percentage of identity between the sequences.

The similarity amongst the BGCs encoded in the *Pseudovibrio* genomes was investigated by performing a global alignment of the concatenated biosynthetic proteins extracted from antiSMASH predictions. Results were then visualized through a similarity network (**Figure 2**), where nodes (representing a BGC) are connected by edges colored according to the percent identity shared between the BGCs. The thickness of the edges is proportional to the coverage index (which was calculated as; the ratio between the lengths of the alignment minus the gaps, divided by the length of the longest sequence in the alignment). We observed overall, a high degree of identity (>75%) between the bacteriocin, NRPS, T1–T3 PKS and terpene BGCs amongst the majority of the *Pseudovibrio* isolates (**Figure 2**). Major variability was observed amongst the class of hybrid NRPS-PKS and T3 PKS. These implied relationships were found to be in agreement with 16S rRNA phylogenetic analysis of the isolates wherein nodes representing >75% identity among isolates belonged to *Pseudovibrio* strains which branched closely together on the 16S tree (**Figures 1**, **2**). Consistent with the 16S based phylogeny, the most dissimilar BGCs were identified in *P. axinellae* AD2, *Pseudovibrio* sp. Tun.PHSC04-5.I4 and *P. stylochi* MCCC 1K00452 (UST20140214-052). These isolates formed independent branches on the 16S phylogenetic tree (**Figure 1**). The similarity network generated for analysis of the siderophore BGCs is a good example which highlights this finding (**Figure 2**). We see the node representing the cluster from *P. axinellae* AD2 is clearly distant from the other related BGCs, sharing limited similarity only with the clusters of strain AD5, 13, 37, and 46 (**Figure 2**).

**FIGURE 3:**
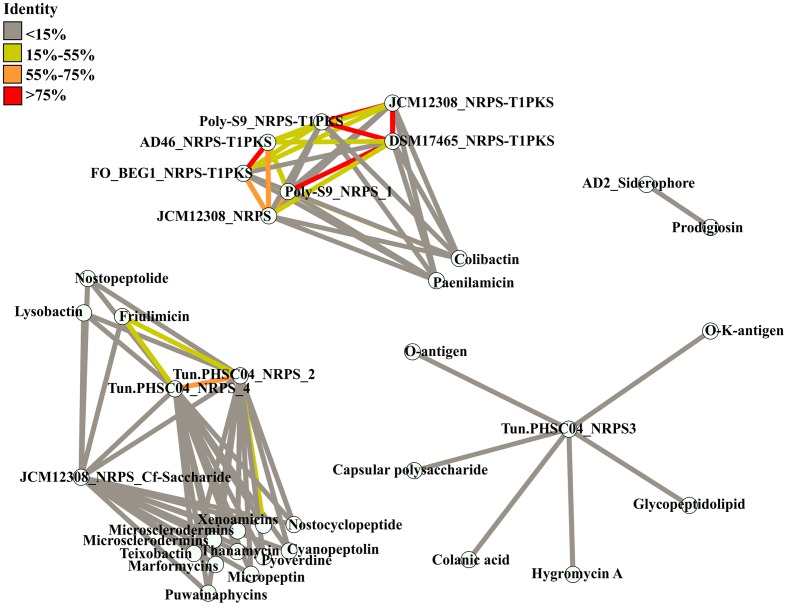
Similarity between BGCs detected in the *Pseudovibrio* genomes and known BGCs. Known BGCs showing the most similarity to those detected in the *Pseudovibrio* following antiSMASH analysis were selected and the amino acid sequences of the associated biosynthetic genes were aligned with those of the *Pseudovibrio* clusters. Each node represents concatenated amino acid sequences. Edges are colored according to the percentage of identity shared between the clusters.

It is interesting to point out the uneven distribution of the T3-PKS and the hybrid NRPS-PKS, together with the limited similarity that most of these BGCs shared (**Figure 2**). Finally, it is worth noting the multiplication of NRPS and PKS-like BGCs observed in *Pseudovibrio* sp. Tun.PHSC04-5.I4 (Lake Vida isolate; hypersaline, extreme cold environment) and the limited similarity shared amongst them, together with the complete absence of such BGCs categories in the genomes of both flatworm isolates, *P. hongkongenesis* MCCC 1K00451 (UST20140214-015B) and *P. stylochi* MCCC 1K00452 (UST20140214-052) (**Figure 2**). Where applicable (**Figures 2**, **3**), *P. stylochi* is referred to as Pstyl, *P. hongkongenesis* as Phongk and *Pseudovibrio* sp. Tun.PHSC04-5.I4 as Tun.PHSC04 for ease of reference.

### NRPS Clusters

13 out of the 21 *Pseudovibrio* isolates examined contained at least 1 NRPS cluster, this included all 10 *A. dissimilis* (AD) isolates (1 NRPS cluster each), the seawater isolate *P. denitrificans* JCM12308 (1 NRPS cluster), *Polymastia penicillus* isolate POLY-S9 (2 NRPS clusters) and the Lake Vida isolate *Pseudovibrio* sp. Tun.PHSC04-5.I4 (4 NRPS clusters). The clusters ranged in size from 26.8 kb (POLY-S9) to 79.9 kb [W64 (AD8)]. Some variability in domain organization was observed amongst the *Pseudovibrio* NRPS BGCs. The NRPS clusters identified in 5 out of 10 *A. dissimilis* isolates [AD2, AD13, AD14, W74 (AD15) and AD37] shared the same domain organization (**Supplementary Table S2A**) consisting of five modules containing the classical C-A-T tridomain architecture associated with NRPS clusters, a 6th module containing a heterocyclization domain, a 7th module containing the classical C-A-T tridomain architecture, and a termination Te domain. A series of binding and condensation domains as well as a keto synthase (KS), keto reductase (KR), and a thioesterase (Te) domain also formed part of the NRPS biosynthetic machinery (**Supplementary Table S2A**). Although similarities existed, this domain arrangement did not meet the requirements necessary to be considered as a PKS module, since it lacked AT and ACP domains. Two additional genes containing an ACPS domain and a Te domain were also determined to be part of these clusters (**Supplementary Table S2A**). antiSMASH uses (i) NRPSPredictor2^2^ (ii) the method of Minowa et al. (2007) and (iii) the specificity conferring code proposed by Stachelhaus et al. (1999) to predict the consensus substrate which binds at the A domain in NRPS clusters. All predictions are then integrated into a consensus prediction by majority vote. Based on the binding specificities of the A domains associated with the aforementioned NRPS clusters, the amino acid backbone structure of the potential peptide was predicted as: Gly-Thr-Tyr-Cys-Pro-Arg-x-Pro-x, where ‘x’ represents an unknown residue (**Table 2**).

**Table 2:** Predicted amino acid backbone structure of non-ribosomal peptide synthetases (NRPS) and NRPS-PKS hybrid clusters.

Strain	BGC	Predicted amino acid backbone structure
***Pseudovibrio* sp.**		
AD2, AD13, AD14, W74	NRPS	Gly-Thr-Tyr-Cys-Pro-Arg-x-Pro-x
AD5, AD46, POLY-S9 (2)	NRPS	Tyr-Thr-Gly-Arg-Pro-Cys-x-x-Pro
AD37, WM33	NRPS	Tyr-Thr-Gly-Arg-Pro-Cys-Gln-x-Pro
W64	NRPS	Tyr-Thr-Gly-Arg-Pro-Cys-x-x-Pro-Leu-Ala-x
AD26	NRPS	x-Thr -Phe-Cys-Pro-Arg-Gln-Pro-x
Tun.PHSC04-5.I4 (1)	NRPS	Gly-Thr-Tyr-Gly-Cys-Pro-Arg-x- Pro-Val
Tun.PHSC04-5.I4 (2)	NRPS	Gly-x-x-x-Asn-Gly
Tun.PHSC04-5.I4 (3)	NRPS	Tyr-Asn-x-Asn-Gly-Gly-x
Tun.PHSC04-5.I4 (4)	NRPS	Gly-Asn-Asn-x
FO-BEG1	NRPS-PKS	Val-Ser-x-Gly-Cys-Cys-Asn
AD46	NRPS-PKS	Val-x-Ser-Cys-Gly-Asn
POLY-S9	NRPS-PKS	Val-Ser-x
***P. denitrificans***		
DSM17465	NRPS-PKS	Val-Ser-x-Gly-Cys-Cys-Asn
JCM12308	NRPS-PKS	Val-x

*Where noted, ‘*x*’ represents an unknown residue*.

The NRPS clusters identified in *Pseudovibrio* sp. AD5 and AD46 demonstrated the same module architecture as described in the aforementioned isolates but additionally contained a polyketide cyclase and an enoyl-CoA hydratase domain (**Supplementary Table S2A**). Analysis of the binding specificities of the A domains in these NRPS clusters resulted in a predicted backbone structure consisting of Tyr-Thr-Gly-Arg-Pro-Cys-x-x-Pro amino acid residues (**Table 2**). NRPS derived peptides in *Pseudovibrio* sp. WM33 (AD30) and POLY-S9 were determined to possess a Tyr-Thr-Gly-Arg-Pro-Cys-Gly-x-Pro amino acid backbone structure (**Table 2**), while *Pseudovibrio* sp. W64 (AD8) possesses a peptide with a predicted structure of Tyr-Thr-Gly-Arg-Pro-Cys-x-x-Pro-Leu-Ala-x residues (**Table 2**). Three additional AMP binding domains were located at the C terminus of the predicted peptide in this strain (**Supplementary Table S2A**). An additional ACPS domain and a PCP domain were located toward the C terminus of the peptide in *Pseudovibrio* sp. AD26 (**Supplementary Table S2A**). The predicted structure of the peptide produced from the NRPS cluster in this strain is; x-Thr-Phe-Cys-Pro-Arg-Gln-Pro-x (**Table 2**).

Each of the four NRPS clusters found in *Pseudovibrio* sp. Tun.PHSC04-5.I4 differed in the total number and organization of their domains (**Supplementary Table S2A**) as well as the predicted amino acid backbone structure of the molecules derived from them. NRPS cluster 1 from this organism consisted of 32 domains with a predicted backbone structure of Gly-Thr-Tyr-Gly-Cys-Pro-Arg-x-Pro-Val, NRPS cluster 2 contained 20 domains and a predicted amino acid backbone structure of Gly-x-x-x-Asn-Gly (**Table 2**). NRPS cluster 3 consisted of 23 domains, one of which was determined to be an epimerization domain (which may function in epimerizing the innermost amino acid of the peptide chain to a D-configuration) (**Supplementary Table S2A**). The amino acid backbone structure produced by the latter was predicted to be Tyr-Asn-Nrp-Asn-Gly-Gly-x. Finally, NRPS cluster 4 contained 18 domains, with a predicted structure of Gly-Asn-Asn-x (**Table 2**). In addition, a large number of mobile genetic elements were found in association with these NRPS clusters in *Pseudovibrio* sp. Tun.PHSC04-5.I4. NRPS cluster 3 in particular was bookended by integrases and transposases (ORF_05677-05680, ORF_05686, ORF_05689, ORF_05693; **Supplementary Table S1**).

The majority of the NRPS clusters identified amongst the *Pseudovibrio* showed no homology to known BGCs and differed in the number, arrangement and potential function of the tailoring, transport and regulatory genes associated with them (**Supplementary Table S1**). According to the antiSMASH analysis, only a small percentage of genes belonging to the NRPS clusters of *P. denitrificans* JCM12308, *Pseudovibrio* sp. Tun.PHSC04-5.I4 (NRPS clusters 2, 3, and 4) and *Pseudovibrio* sp. POLY-S9 (NRPS cluster 1) showed similarity to known homologous gene clusters (**Supplementary Table S3**). It is therefore likely that the natural products derived from these gene clusters are novel compounds with diverse chemical structures.

### PKS Clusters

Polyketide synthase BGCs were identified in 19 out of the 21 *Pseudovibrio* strains examined. *Pseudovibrio* sp. JCM19062 and Tun.PHSC04-5.I4 were the only isolates to contain both type I (T1) and type III (T3) PKS clusters, with JCM19062 containing one of each PKS cluster type and Tun.PHSC04-5.I4 containing two of each PKS cluster type. *P. axinellae* AD2 and *Pseudovibrio* sp. POLY-S9 were also found to contain a T3 PKS cluster. T1 PKS clusters ranged in size from 13.8 kb (JCM19062) to 51 kb (Tun.PHSC04-5.I4), while T3 PKS clusters ranged in size from 6.3 kb (JCM19062) to 63.3 kb (JE062). The T1 and T3 PKS systems differed in the number and type of tailoring, transport and regulatory genes associated with them (**Supplementary Table S1**) and demonstrated no homology to known BGCs, suggesting that the compounds produced from these clusters may be novel. T1–T3 PKS clusters were identified in all remaining isolates [with the exception of the flatworm isolates, *P. hongkongenesis* MCCC 1K00451 (UST20140214-015B) and *P. stylochi* MCCC 1K00452 (UST20140214-052)]. These T1–T3 PKS clusters appeared relatively homologous amongst the *Pseudovibrio* isolates in which they were located, sharing many biosynthetic, tailoring, regulatory and transport elements (**Supplementary Table S1**). The domain organization for the T1–T3 PKS clusters was the same in all isolates, consisting of the essential ketosynthase (KS) domain, AT domain and ACP domains synonymous with PKS systems. Each T1–T3 PKS system additionally contained two KR domains, a DH, an enoyl-reductase (ER) and an aminotransferase domain which function to contribute to the overall structural diversity of the resulting product (**Supplementary Table S2D**). A PKS docking domain was additionally located at the C terminus of the peptide in *Pseudovibrio* sp. FO-BEG1. As was previously observed for the T1 and T3 PKS clusters, these T1–T3 PKS clusters demonstrate no homology to known BGC’s.

### NRPS-PKS Hybrid Clusters

*Pseudovibrio* sp. AD46, FO-BEG1, POLY-S9 and *P. denitrificans* DSM17465 and JCM12308 each contained an NPRS-T1PKS hybrid cluster. Hybrid systems are of particular interest to researchers as high levels of biosynthetic potential can be derived from a combination of NRPS and PKS activities, resulting in a large variety of structures and diverse chemical compounds (Liu et al., 2004; Mattheus et al., 2010; Park et al., 2010). The NRPS-PKS hybrid cluster of *P. denitrificans* DSM17465 was determined to be the largest amongst those identified at 93 kb, followed by that of *Pseudovibrio* sp. FO-BEG1 at 83.9 kb. The hybrid cluster identified in *Pseudovibrio* sp. AD46 is 66 kb, while that of *P. denitrificans* JCM12308 is 47.8 kb. The NRPS-PKS hybrid cluster of *Pseudovibrio* sp. POLY-S9 was determined to be the smallest of those identified at 28.8 kb.

The domain organization and overall module architecture of the core biosynthetic genes of the NRPS-PKS hybrid clusters located in *Pseudovibrio* sp. FO-BEG1 and *P. denitrificans* DSM17465 was identical (**Supplementary Table S2B**) as well as the final predicted amino acid backbone structure of Val-Ser-x-Gly-Cys-Cys-Asn (**Table 2**). It is worthy to note the presence of two heterocyclization domains within the continuity of these hybrid clusters, as they are likely to catalyze the formation of heterocyclic rings from the cysteine residues predicted to be bound by the A domains which precede them (**Supplementary Table S2B**), thereby adding to the structural diversity of the molecules potentially produced by these organisms.

The NRPS-PKS hybrid cluster located in *Pseudovibrio* sp. AD46 closely resembled that of *Pseudovibrio* sp. FO-BEG1 and *P. denitrificans* DSM17465 in domain organization. However, the cluster lacked five domains associated with the hybrid clusters from the aforementioned strains (i.e., 2 × PCP, 1 × KS, heterocyclization and an AMP-binding domain), resulting in a predicted amino acid backbone structure consisting of Val-x-Ser-Cys-Gly-Asn residues (**Supplementary Table S2B** and **Table 2**).

The NRPS-PKS hybrid clusters associated with *P. denitrificans* JCM12308 and *Pseudovibrio* sp. POLY-S9 were found to lack many of the domains associated with the same cluster type in *Pseudovibrio* sp. FO-BEG1 and *P. denitrificans* DSM17465 including the heterocylization domains, but showed overall a high degree of similarity to each other in module organization (**Supplementary Table S2B**). An additional condensation domain was observed at the N terminus of the cluster in *P. denitrificans* JCM12308 while *Pseudovibrio* sp. POLY-S9 contains an additional AMP binding domain preceding the NRPS module of the cluster (**Supplementary Table S2B**). The resulting amino acid structures predicted were Val-x for the hybrid cluster identified in *P. denitrificans* JCM12308 and Val-Ser-x for the cluster identified in *Pseudovibrio* sp. POLY-S9 (**Table 2**).

As well as the differences observed at the domain level, the NRPS-PKS hybrid clusters differed in the number and type of tailoring, transport and regulatory genes associated with them (**Supplementary Table S1**), thus adding further potential toward the production of structurally diverse compounds from these isolates.

### Similarity between *Pseudovibrio* BGCs and Other Characterized BGCs

antiSMASH analysis revealed that only a minority of the BGCs identified in the *Pseudovibrio* genomes share similarity with known and characterized BGCs and where present, this similarity was low overall (**Supplementary Table S2**). In general, most of the hybrid NRPS-PKS clusters identified in the *Pseudovibrio* genomes, showed a variable degree of similarity with the BGC responsible for colibactin production. According to antiSMASH, 26% of genes located within the colibactin gene cluster showed similarity with genes located in the NRPS-PKS hybrid clusters of *P. denitrificans* DSM17465, *Pseudovibrio* sp. AD46 and FO-BEG1, 21% showed similarity with those located in the hybrid NRPS-PKS cluster of *P. denitrificans* JCM12308, and 17% shared similarity with genes within the *Pseudovibrio* sp. POLY-S9 NRPS-PKS hybrid cluster (**Supplementary Table S3**). Similarly, antiSMASH revealed that 42% of genes located within the BGC responsible for the production of xenoamicin (a peptide antibiotic produced by the genus *Xenorhabdus*; Zhou et al., 2013) showed similarity with genes located within NRPS cluster 2 from *Pseudovibrio* sp. Tun.PHSC04-5.I4. However, we determined this was due to the similarity of multiple genes with one single ORF (ORF_05633) within the BGC of Tun.PHSC04-5.I4.

The overall low similarity which exists between the BGCs identified in the *Pseudovibrio* isolates and those characterized in other bacteria was confirmed following a global alignment of the amino acid sequences of all biosynthetic proteins involved in secondary metabolite production in the *Pseudovibrio* BGCs with those identified by antiSMASH as being the most similar clusters (**Figure 3**). The greatest identity observed was just 17.2%, between the BGC involved in the synthesis of friulimicin (a lipopeptide antibiotic produced by *Actinoplanes friuliensis*; Muller et al., 2007) and NRPS cluster 4 from Tun.PHSC04-5.I4. The *Pseudovibrio* NRPS and NRPS-PKS hybrid clusters which antiSMASH determined as sharing some similarity to a colibactin gene cluster, in fact shared limited similarity with the colibactin cluster in the alignment we performed. The highest identity value observed was just 16.9% and was determined to be between NRPS cluster 1 from strain POLY-S9 and the colibactin BGC (**Figure 3**).

**FIGURE 4:**
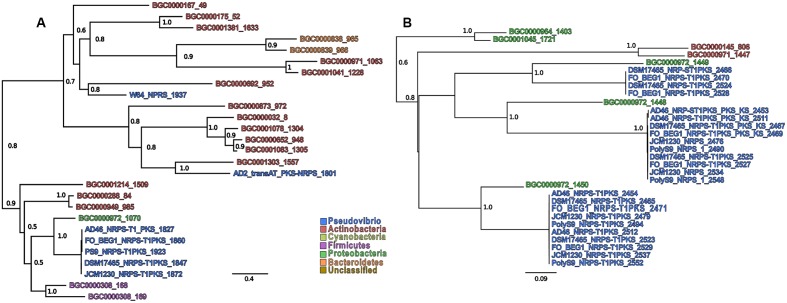
Sub-tree containing *Pseudovibrio* sequences extracted from **(A)** the AMP-binding domains and **(B)** the KS domains. Only support values higher than 0.5 are shown. The numbers after the BGCs names refer to the position of the sequence in the alignment. Due to the presence of multiple domains in the same BGCs, each sequence was numbered in order to improve clarity.

In order to infer both evolutionary and functional homology between the NRPS, PKS, and NRPS-PKS BGCs in the *Pseudovibrio* genomes and those located in the MIBiG repository, we performed a phylogenetic reconstruction using the AMP and KS binding domains associated with these BGCs (detected by antiSMASH) against all entries in the MIBiG database (**Figures 4A,B** and Supplementary Figures S1–S4). We determined that the A and KS domains located within the *Pseudovibrio* NRPS-PKS hybrid clusters formed unique branches (whilst remaining well separated) with those identified in the gene cluster for colibactin synthesis (BGC000972; **Figures 4A,B** and Supplementary Figures S3C,D,H,I, S4C). The A domains identified in the remaining (i) NRPS clusters (ii) NRPS_Cf-saccharide clusters (iii) the *trans*-AT-PKS-NRPS hybrid cluster of *P. axinellae* AD2 formed quite independent branches in the tree, relating mostly with actinomycetes and gammaproteobacterial sequences. Finally, the domains identified in NRPS clusters 2–4 in strain Tun.PHSC04-5.I4 branched together with domains from clusters belonging to the deltaproteobacteria order of the *Myxococcales* (Supplementary Figure S3G).

Overall, the KS domains showed the most heterogeneity in terms of phylogenetic relationship (**Figure 4B**). The domains belonging to the *trans*-AT PKS-NRPS hybrid cluster from strain AD2 were the most dissimilar among those investigated, frequently forming independent, well-defined branches on the phylogenetic trees (Supplementary Figures S4E,F). In agreement with the phylogenetic reconstruction performed for the AMP binding domains, all the KS domains identified within the *Pseudovibrio* hybrid NRPS-PKS systems, clustered together with domains belonging to the colibactin gene cluster, remaining, however, well separated from the latter. The rest of the KS domains (mainly those belonging to the T1–T3 PKS cluster types) showed a high degree of similarity within the genus. The most dissimilar KS domains were those obtained from sequences belonging to strain Tun.PHSC04-5.I4. It should be noted that the phylogenetic reconstruction performed in this study is based on the sequences of characterized BGCs available in the MIBiG database, in which some bacterial taxa are under-represented. Thus, the analyses we report here aims to compare the as yet undescribed *Pseudovibrio* sequences with other BGCs of biotechnological interest, and should not be interpreted as a comprehensive analysis aimed to elucidate the phylogenetic history of the PKS and NRPS gene clusters found in this genus.

## Discussion

Members of the *Pseudovibrio* genus are frequently the most abundant isolates recovered from amongst the bacterial fraction of marine invertebrates, particularly sponges, and have been shown to demonstrate a wide spectrum of inhibitory properties against prominent human pathogens (O’Halloran et al., 2011; Esteves et al., 2013; Bauvais et al., 2015). However, to-date, little information exists in relation to the biosynthetic capabilities of these organisms or the assortment of secondary metabolites which they potentially produce. In this work, we present for the first time, a comprehensive analysis of the distribution and diversity of the BGCs in the available *Pseudovibrio* genomes, based on comparative genomics. This approach is becoming increasingly more relevant as a fast and inexpensive approach for the identification of “talented” bacteria.

One of the first aims of this study was to identify the redundancy of BGCs within the genus and their similarity with known and characterized biosynthetic clusters. In order to avoid the known problem of border definition in BGCs (Cimermancic et al., 2014) we decided to use a conservative approach to explore the similarity of the BGCs (i) within the *Pseudovibrio* genus itself and (ii) between the *Pseudovibrio* genus and other bacteria. We performed global alignments of the amino acid sequences of the proteins predicted by antiSMASH to be involved in the biosynthesis of secondary metabolites and visualized the results via a similarity network. These analyses revealed that for some families (bacteriocin, NPRS, T1-T3PKS, terpene) the overall identity of the BGCs within the genus is considerably high (> = 75%; **Figure 2**). Given that the majority of the *Pseudovibrio* strains investigated have been isolated from marine invertebrates, it is tempting to speculate that these metabolites could play a role in interactions with the host or be used by *Pseudovibrio* to interact with the rest of the bacterial community while establishing itself in the host microbiome, as has been previously described for other bacteria and fungi (Sharon et al., 2014; Sbaraini et al., 2016). However, some significant degree of variability existed among the strains. For example, clusters synthesizing aryl polyene-like molecules were found in only 2 out of 21 strains (**Figure 1**). These molecules are a class of natural product, previously described as pigments and more recently as possessing antioxidant properties (Cimermancic et al., 2014; Schoner et al., 2016). Interestingly, derivatives of aromatic polyene have been previously isolated from marine organisms and shown to possess antibacterial and antitumor activities (Kwon et al., 2006). Such a finding emphasizes the significance of chemical characterization of the molecules produced by *Pseudovibrio* isolates.

Particularly intriguing is the presence of a hybrid *trans*-AT PK-NRPS gene cluster only in the type strain *P. axinellae* AD2. *trans*-AT PKS systems are involved in the biosynthesis of many pharmacologically important polyketides, such as the antibiotic mupirocin (El-Sayed et al., 2003) and lankacidin, which possesses antitumor activities (Kende et al., 1995). This type of PKS differs from the canonical PKS (also called “*cis* AT-PKS”) because it has free-standing AT domains, as opposed to the integrated AT domains found within the modules of *cis* AT-PK multienzyme systems (Piel, 2010). Phylogenetic analysis revealed that the KS domains of this *trans*-AT PK-NRPS BGC in AD2 formed independent branches, with good statistical support (Supplementary Figures S4E,F). An antiSMASH comparison revealed 61% similarity between the *trans*-AT PK-NRPS BGC of strain AD2 and a gene cluster from *Xenorhabdus bovienii* str. oregonense, belonging to a genus of Gammaproteobacteria, well known for its ability to produce bioactive secondary metabolites (Bode, 2009). These findings suggest that the *trans*-AT PKS-NRP gene cluster may have been acquired by strain AD2 via horizontal gene transfer (HGT). Consistent with these results, several lines of evidence indicate that *trans*-AT PKS genes are DNA mosaics assembled from multiple fragments by means of extensive HGT (Nguyen et al., 2008). Interestingly, most of the time *trans*-AT PKS-related gene clusters not of actinomycete origin are found in symbiotic or pathogenic bacteria, suggesting that for an as yet unspecified reason, the metabolites produced from these BGC types, may play an important role in microbe-host interactions (Piel, 2010). In line with these findings, strain AD2 was isolated from the sponge *A. dissimilis* and previous comparative genomic studies suggested that this strain might represent an ancient symbiont of marine sponges, having lost many mechanisms that would be required by bacteria to colonize the host (Romano et al., 2016). Altogether, these findings suggest that strain AD2 is a “talented” strain that could be a prolific source of bioactive natural products.

Even though some gene cluster types were shared among many *Pseudovibrio* strains (i.e., NRPS), a more detailed analysis of the domain organization of these BGCs revealed a certain degree of specificity. Many of the NRPS clusters in particular, differed in the number, organization and function of their associated domains, indicating a potential for variation in the final structure of the resulting products (**Supplementary Table S2A**). NRPS product diversity derives primarily from the substrates incorporated at the adenylation (A) domain in each module. For example, iturin, mycosubtilin and bacillomycin are related molecules produced by *Bacillus* NRPSs. The first halves of these NRPS clusters have the same A domain organization, and as a result, the first ‘halves’ of these molecules are structurally the same. However, differences in the A domain substrate selectivity in the second halves of these NRPS clusters leads to incorporation of different building blocks at these domains, ultimately resulting in the latter part of the iturin, mycosubtilin, and bacillomycin molecules being structurally different (Moyne et al., 2004). Analysis of the predicted A domain binding specificities in the NRPS clusters identified in this study highlighted potential for the production of a number of structurally diverse novel products from members of the *Pseudovibrio* genus (**Table 2**).

Considering that most of the *Pseudovibrio* strains were isolated from marine invertebrates, it is tempting to speculate that some of the secondary metabolites produced may play a role in interactions with the eukaryotic host. It is therefore reasonable to imply that these strains may represent a source of novel bioactive compounds, not only with antibacterial properties but also potentially with direct effects on eukaryotic systems. This hypothesis is strengthened by the recent analyses performed in other bacteria on the colibactin-like gene cluster. Colibactin is a bacterial toxin, whose structure is still unknown. It has been shown to play a role in the virulence of *E. coli* by inducing DNA double-strand breaks in eukaryotic cells (Nougayrede et al., 2006). The hybrid NRPS-PKS gene cluster responsible for the production of colibactin and its precursor has long been associated with the Enterobacteriacea, encoding genes involved in the production of a variety of molecules important in colon cancer development (Brachmann et al., 2015; Li et al., 2015). Recently, similar BGCs have also been identified in *Pseudovibrio* sp. FO-BEG1 and in *Frischella perrara* PEB0191, a bacterium that colonizes the honey bee gut (Bondarev et al., 2013; Engel et al., 2015). Additionally, it has been demonstrated that like *E. coli, F. perrara* causes DNA damage in eukaryotic cells *in vitro* in a colibactin pathway-dependent manner (Engel et al., 2015). Taken together therefore, it seems reasonable to speculate that bacteria belonging to the *Pseudovibrio* genus are capable of producing colibactin-like molecules which could have valuable medical and biotechnological applications. The low level of identity observed between the amino acid sequences and the hybrid NRPS-PKS of *Pseudovibrio* and the colibactin gene cluster, together with the phylogenetic separation between the KS and AMP domains (**Figures 3**, **4A,B** and Supplementary Figures S3, S4), indicates a certain degree of divergence between the colibactin-like clusters in *Pseudovibrio* and the one characteristic of enterobacteria. Moreover, some variability in the accessory biosynthetic genes exist between the MIBiG colibactin and the hybrid NRPS-PKS BGC in the *Pseudovibrio*. For example, in *Pseudovibrio* sp. FO-BEG1, additional genes encoding proteins involved in transamination and glutamate metabolism (ORF_03314 and ORF_03316) might be potentially involved in the final maturation of the molecule produced by this cluster. This suggests that the final product of the hybrid NRPS-PKS in FO-BEG1 might differ from the one synthesized by the colibactin gene cluster.

Biosynthetic gene clusters or parts of them have been shown to be subject to extensive HGT (Ginolhac et al., 2005; Ziemert et al., 2014). The phylogenetic analysis we performed offers a clear indication that in some cases, events of HGT transfer are likely to have led to the acquisition of some BGCs in *Pseudovibrio* strains (Supplementary Figures S1–S4). A few interesting examples are offered by the aforementioned *trans*-AT PKS-NRPS BGC, the hybrid NRPS-PKS found in a fraction of the strains, and the multiple NRPS clusters observed in the genome of strain Tun.PHSC04-5.I4 (which branched in the tree with sequences belonging to order Myxococcales). In the latter case the presence of multiple mobile elements clearly indicates that the whole region encoding for the multienzyme system might have gone through multiple dislocation events and rearrangement in the genome of the strain.

It is important to point out that a screening of the annotated *Pseudovibrio* genomes revealed the presence of additional genes involved in polyketide synthesis and maturation which were not detected in the antiSMASH analysis (**Supplementary Table S4**). One of the most interesting proteins encoded by these genes was annotated as polyketide cyclase SnoaL-like or containing the SnoaL-domain. We verified the presence of this domain by scanning all the proteins via Interpro, and in all cases these proteins either contained the polyketide cyclase SnoaL-like domain (IPR009959), or the parent domain NTF2-like (IPR032710) (**Supplementary Table S4**). Genes encoding proteins containing the same domains were detected in the T3 PKS cluster 2 in strain Tun.PHSC04-5.I4, in the T1 PKS-bacteriocin cluster of strain JCM1230 and DSM17465, and in the NRPS cluster of strain AD2 (**Supplementary Tables S1**, **S3**). This enzyme has been well characterized in the *Streptomyces* genus and catalyzes the last ring closure step during the synthesis of several compounds with antibacterial and anticancer properties, such as anthracyclines. These are a group of aromatic polyketide antibiotics, which contain some of the clinically most potent anti-tumor drugs, e.g., doxorubicin and daunorubicin (Strohl et al., 1989; Strohl, 2001). A particular feature associated with this enzyme is its ability to create a cyclic product with specific stereochemical properties of high pharmacological value (Sultana et al., 2004). Finding such a group of enzymes encoded in the *Pseudovibrio* genomes underlines how the variability of the final molecules produced by the BGCs in these bacteria goes beyond the sole identification of the PKS clusters, and suggests once more, the likely presence of pharmaceutically valuable compounds in the secondary metabolome of these organisms. Interestingly, the potential production of condensed molecules is consistent with the untargeted metabolomics approach previously performed, that showed an increase in unsaturated and highly unsaturated molecules in *Pseudovibrio* cultures (Romano et al., 2014).

The low degree of similarity shared between the BGCs identified in the *Pseudovibrio* genomes and those reported in the MIBiG database (detected by antiSMASH analysis and via results observed in the similarity network in **Figure 3**) indicates the potential novelty of the metabolites produced by these *Pseudovibrio*’s pathways. Our analysis is therefore consistent with the broad range of inhibitory properties that have been repeatedly described for *Pseudovibrio* strains (Crowley et al., 2014) wherein, it seems likely that more than one antimicrobial compound is being produced by these strains. The diversity of BGCs we report, and the high antagonistic ability of these strains would justify why *Pseudovibrio* is often the dominant isolate retrieved from the bacterial communities of marine sponges. Altogether, this data calls for a more thorough investigation of the secondary metabolites produced by the *Pseudovibrio*, which may promise novel molecules of biotechnological interest. So far only a limited number of molecules produced by *Pseudovibrio* have been chemically characterized (Sertan-de Guzman et al., 2007; Nicacio et al., 2017). It could be that sensitive bioassays have not yet been developed to facilitate identification of the compounds produced by these bacteria or that most of the BGCs identified in the *Pseudovibrio* genomes are cryptic or “silent” and are not expressed under standard laboratory growth conditions. In order to overcome this problem, different molecular and cultivation based strategies need to be implemented in order to stimulate the expression of such “silent” BGCs. Previous physiological studies have already shown the dramatic effect that nutrient availability has on the pattern of secreted metabolites in some members of the *Pseudovibrio* genus (Romano et al., 2014, 2015, 2017) therefore, in the first place different nutrient regimes and environmental cues need to be explored in order to fully investigate the biosynthetic capabilities of *Pseudovibrio* strains. Secondly, new genetic tools need to be established in order to access the genetic potential of these marine bacteria, since, to-date, these organisms have remained largely recalcitrant to genetic manipulation. Given the diversity of potentially novel bioactive compounds which may be produced by these marine bacteria, we believe these to be worthwhile endeavors in helping to unlock the biosynthetic potential of these talented microorganisms.

## Author Contributions

The study was designed by LMN, SR, FOG, and ADWD. SR performed the comparative genomic analyses with the help of LMN. LMN and SR wrote the manuscript with input from ADWD. All authors reviewed and approved the final version of the manuscript.

## Conflict of Interest Statement

The authors declare that the research was conducted in the absence of any commercial or financial relationships that could be construed as a potential conflict of interest.

## Footnotes

1 http://tree.bio.ed.ac.uk/software/figtree/

2 http://nrps.informatik.uni-tuebingen.de

## Supplementary Material

TABLE S1 BGCs identified in 21 *Pseudovibrio* isolates. Strain designations are indicated on each tab. Relevant information pertaining to BGC type, coordinates, locus tag and gene annotation are included for each strain.

TABLE S2 Domain organization of antiSMASH predicted NRPS clusters **(A)**, NRPSPKS hybrid clusters **(B)**, NRPS-Cf saccharide clusters **(C)** and T1–T3 PKS clusters **(D)**. *Pseudovibrio* strain designations are indicated at the top of each column. Domain organization for the relevant cluster types are outlined accordingly for each strain.

TABLE S3 Similarity of BGCs identified in *Pseudovibrio* sp. to known homologous gene clusters.

TABLE S4 Additional genes involved in polyketide synthesis and maturation which were not detected by antiSMASH analysis.
