# Supplementary material for: Identification of Secondary Metabolite Gene Clusters in the Pseudovibrio Genus Reveals Encouraging Biosynthetic Potential toward the Production of Novel Bioactive Compounds
Source: Front Microbiol. 2017 Aug 18;8:1494. doi: 10.3389/fmicb.2017.01494 (PMC5563371; doi:10.3389/fmicb.2017.01494)
Supplement: Supplementary file 5 [file Presentation_1.PDF]

Supplementary Material

Identification of secondary metabolite gene clusters in the *Pseudovibrio* genus reveals a promising potential toward the discovery of novel bioactive compounds.

Lynn M. Naughton[1$], Stefano Romano [2$], Fergal O'Gara[3,4] and Alan D.W. Dobson[1*]

*** Correspondence:** Professor Alan Dobson: a.dobson@ucc.ie

[$] These authors contributed equally to this work

Supplementary Figure Legends

Figure S1. Unrooted maximum likelihood phylogenetic tree constructed for the AMP-binding domains. The tree includes sequences recovered from the biosynthetic gene clusters obtained from both the MIBiG database and the *Pseudovibrio* genomes.

Figure S2. Unrooted maximum likelihood phylogenetic tree constructed for the KS domains. The tree includes sequences recovered from the biosynthetic gene clusters obtained from both the MIBiG database and the *Pseudovibrio* genomes.

Figure S3. Sub-trees containing *Pseudovibrio* AMP-binding domain sequences. The sub-trees were recovered from the tree shown in Supplementary Figure S1. Only support values higher than 0.5 are shown. The numbers after the BGCs names refer to the position of the sequence in the alignment. Due to the presence of multiple domains in the same BGCs, each sequence was numbered in order to improve clarity.

Figure S4. Sub-trees containing *Pseudovibrio* KS domain sequences. The sub-trees were recovered from the tree shown in Supplementary Figure S2. Only support values higher than 0.5 are shown. The numbers after the BGCs names refer to the position of the sequence in the alignment. Due to the presence of multiple domains in the same BGCs, each sequence was numbered in order to improve clarity.
