# Supplementary figures and images for: Identification of Secondary Metabolite Gene Clusters in the Pseudovibrio Genus Reveals Encouraging Biosynthetic Potential toward the Production of Novel Bioactive Compounds
Source: Front Microbiol. 2017 Aug 18;8:1494. doi: 10.3389/fmicb.2017.01494 (PMC5563371; doi:10.3389/fmicb.2017.01494)

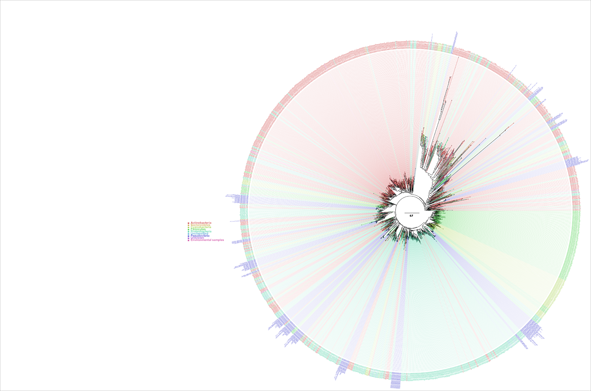

Supplement: Supplementary file 6 [file Image_1.TIF]

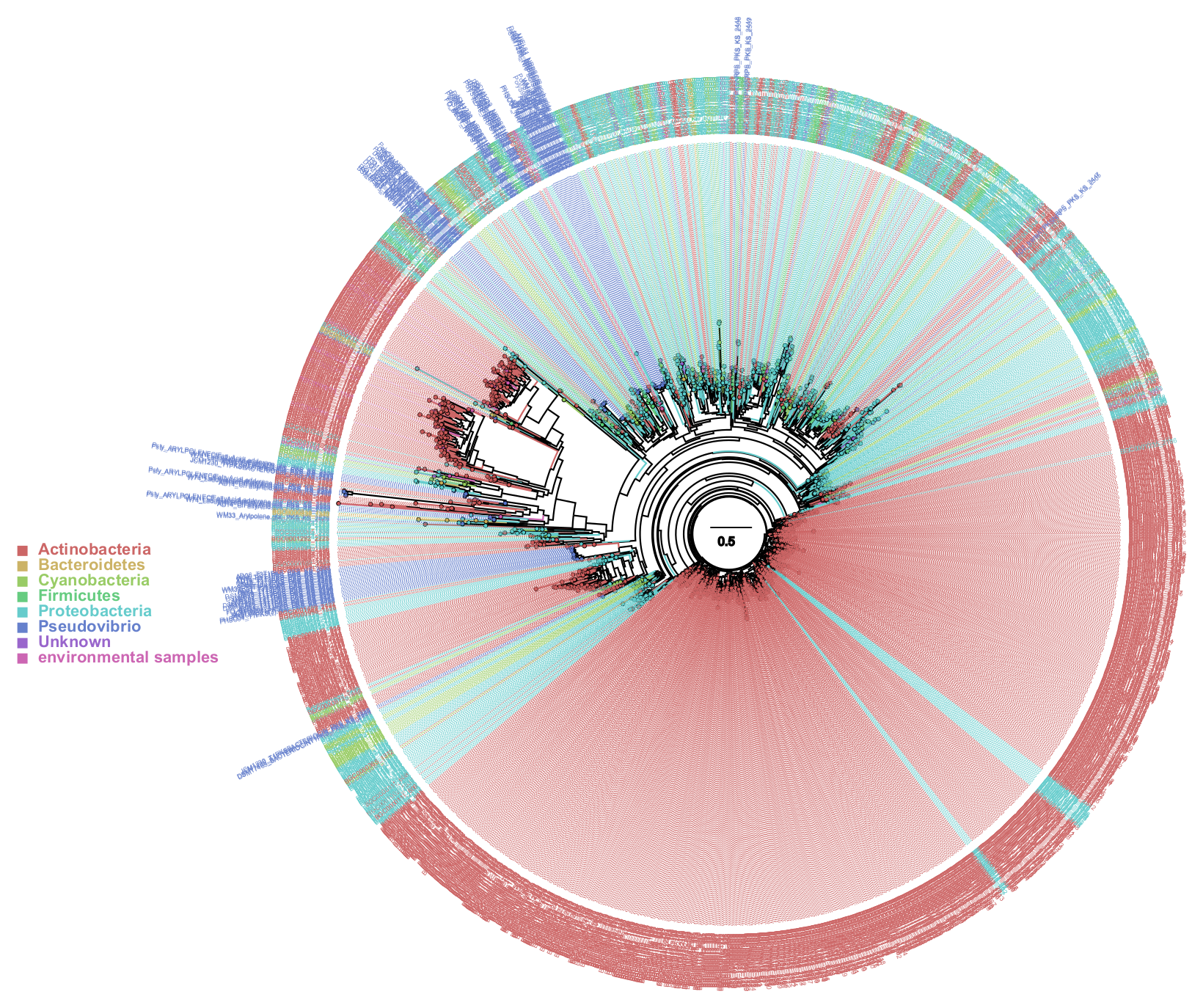

Supplement: Supplementary file 7 [file Image_2.TIF]

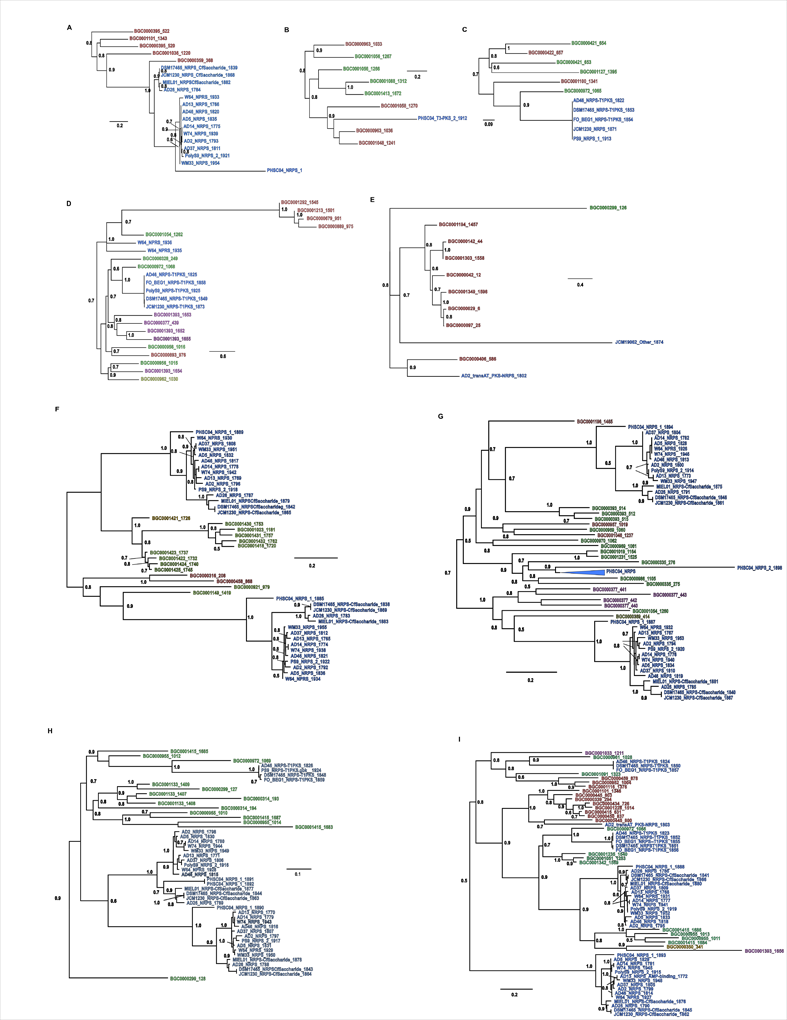

Supplement: Supplementary file 8 [file Image_3.TIF]

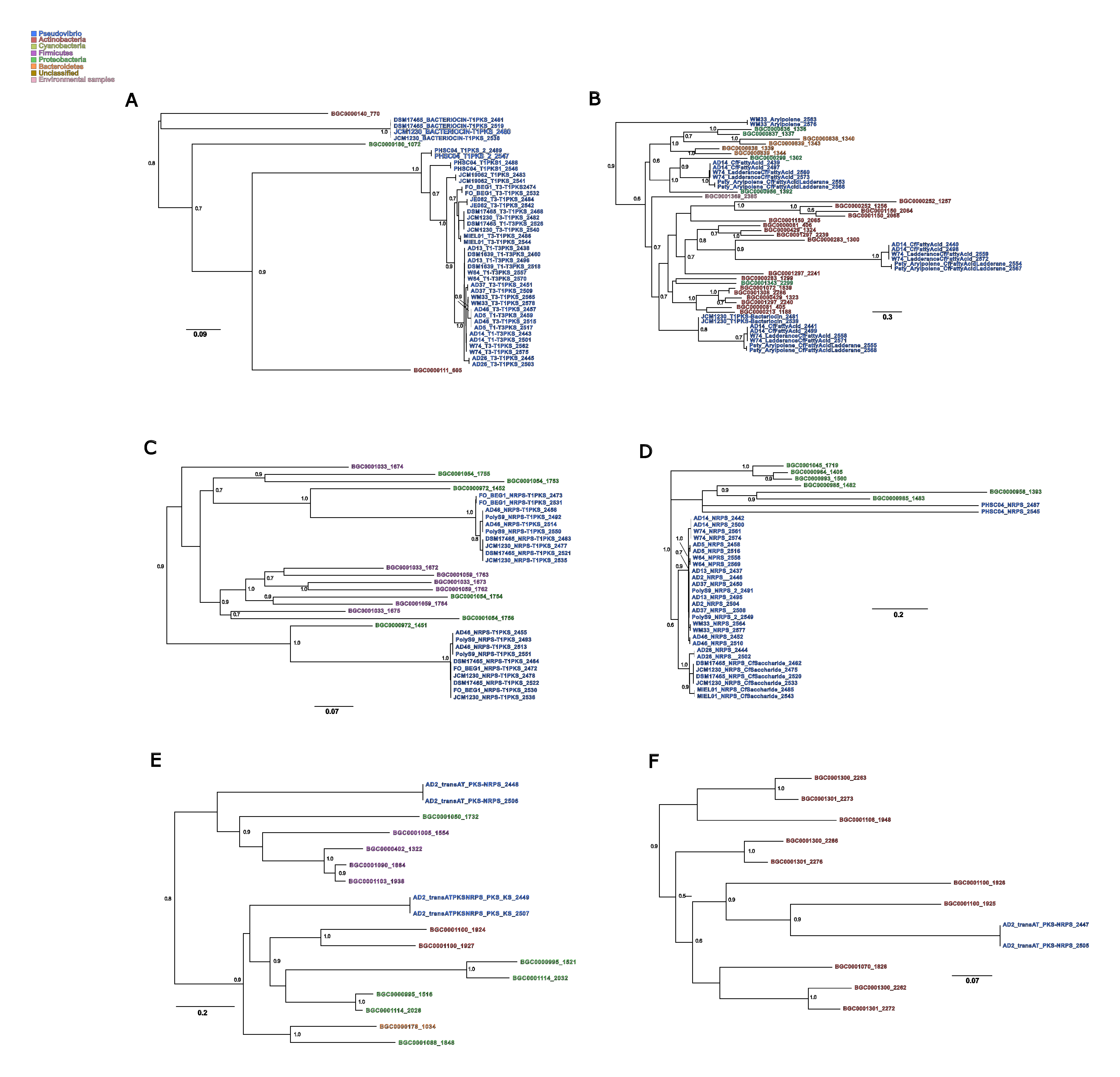

Supplement: Supplementary file 9 [file Image_4.TIF]
